# Supplementary material for: Clinical Implication of Sequential Circulating Tumor DNA Assessments for the Treatment of Diffuse Large B-Cell Lymphoma
Source: Cancers (Basel). 2025 May 22;17(11):1734. doi: 10.3390/cancers17111734 (PMC12153856; doi:10.3390/cancers17111734)
Supplement: Supplementary file 1 [file cancers-17-01734-s001.zip › cancers-3626921-supplementary.pdf]

cancers

MDPI

Article

Clinical Implication of Sequential Circulating Tumor DNA Assessments for the Treatment of Diffuse Large B-Cell Lymphoma

Ga-Young Song [1,†], Joo Heon Park [2,†], Sae-Ryung Kang [3], Seung Jung Han [4], Youjin Jung [4], Minuk Son [4], Ho Cheol Jang [1], Mihee Kim [1], Seo-Yeon Ahn [1], Sung-Hoon Jung [1], Jae-Sook Ahn [1], Je-Jung Lee [1], Hyeoung-Joon Kim [1] and Deok-Hwan Yang [1,*]

[1] Department of Hematology-Oncology, Chonnam National University Hwasun Hospital, Medical School of Chonnam National University, Hwasun-gun 58128, Jeollanam-do, Republic of Korea; songga0e@naver.com (G.-Y.S.); minjokobject@gmail.com (H.C.J.); mihi4300@gmail.com (M.K.); armful12@hanmail.net (S.-Y.A.); shglory@hanmail.net (S.-H.J.); ahnjaesook@hanmail.net (J.-S.A.); drjejung@chonnam.ac.kr (J.-J.L.); hjoonk@chonnam.ac.kr (H.-J.K.)

[2] Department of Laboratory Medicine, Chonnam National University Hwasun Hospital, Medical School of Chonnam National University, Hwasun-gun 58128, Jeollanam-do, Republic of Korea

[3] Department of Nuclear Medicine, Chonnam National University Hwasun Hospital, Medical School of Chonnam National University, Hwasun-gun 58128, Jeollanam-do, Republic of Korea; campanella9@naver.com

[4] Dxome Co. Ltd., Bundang-gu, Seongnam-si 13558, Gyeonggi-do,, Republic of Korea; sjhan@dxome.com (S.J.H.); yjjung@dxome.com (Y.J.); muson@dxome.com (M.S.)

* Correspondence: drydh1685@hotmail.com; Tel.: +82-61-379-7636; Fax: +82-61-7628

† These authors contributed equally to this work.

Supplementary Method

DNA extraction from plasma, peripheral blood mononuclear cells, FFPE (formalin-fixed, paraffin-embedded) and bone marrow mononuclear cells

A total of 18 mL or 9 mL of whole blood was collected in 2 bottles of DxTubeTM cfDNA (Dxome) containing preservation solutions. It was centrifuged at 1900g twice to separate the plasma and buffy coat. Genomic DNA from peripheral blood mononuclear cells (PBMCs) and bone marrow mononuclear cells (BMCs) was extracted using the QIAamp DNA Mini Kit (QIAGEN). Genomic DNA from FFPE (formalin-fixed, paraffin-embedded) was extracted using the QIAamp DNA FFPE Advanced UNG Kit (QIAGEN). Extraction of cfDNA in plasma was performed using magnetic circulating DNA Maxi Reagent (Dxome). Size and quantitative measurements were performed using the D1000 ScreenTape system (Agilent). The concentration of isolated DNA was measured using the Qubit HS dsDNA kit (Invitrogen).

NGS library construction and sequencing

Thirty nanograms of cfDNA were used for experiments, and the entire DNA extract was used for library preparation in clinical samples <30 ng. Library preparation was performed using the DxSeq Lymphoma & Myeloma ctDNA, which includes 112 lymphoma & myeloma-related genes for the Illumina Platform Kit (Dxome) according to the manufacturer's instructions.

Two hundred nanograms of gDNA were used for experiments. Library preparation was performed using the DxSeq Lymphoma & Myeloma PANEL, according to the manufacturer's instructions.

Paired-end sequencing was performed on the NovaSeq 6000 System (Illumina) with a 300-cycle protocol and targeting at least 150 million sequencing reads and >28,000× average sequencing depth per sample. For each sample, PBMCs were sequenced as germline paired controls targeting average sequencing depth of >2,500×. FFPE and BMCs were also sequenced targeting average sequencing depth of >2,500×.

 https://doi.org/10.3390/cancers17111734

Data processing and variant calling

FASTQ data were mapped to the reference genome of GRCh37 (hg19) using the Burrows-Wheeler alignment(1). SNVs, small indels, CNV, and Fusion were called using the PiSeq algorithm (Dxome), which refined the accuracy of molecular barcoding by calculating the genome positions of mapped reads. ExomeDepth was used to cross-check our in-house pipeline for CNV detection(2).

Also, Delly was used to cross-check our in-house pipeline and was performed paired-ends, split-reads, and read-depth to sensitively and accurately delineate genomic rearrangements throughout the genome(2).

Both germline and somatic variants were referred to as matched PBMC and cfDNA samples, respectively. Variants were annotated using the DxSeq software (Dxome), which incorporates public databases for genomic variations. The software automatically produced preliminary classifications of somatic variants according to the Association of Molecular Pathology (AMP), American Society of Clinical Oncology (ASCO), and College of American Pathologists (CAP) standards and guidelines(3) as well as germline variants according to the American College of Medical Genetics (ACMG) and Genomics and the AMP standards and guidelines(4).

Variant interpretation and clinical significance, ctDNA Quantification

Variants including CNV classified as pathogenic, likely pathogenic, or of unknown significance according to the ACMG/AMP guidelines and/or tiers 1, 2, or 3 according to the AMP/ASCO/CAP guidelines were visually inspected using the Integrative Genome Viewer (Broad Institute, USA) to exclude false positives. Variants in both cfDNA and PBMC were determined as germline variants or variants of clonal hematopoiesis of indeterminate potential (CHIP). The ctDNA concentrations were expressed in haploid genome equivalents per mL of plasma (hGE/mL) and calculated by multiplying the mean variant allelic frequency (VAF) for all mutations used for detection calling by the concentration of ctDNA (pg/mL of plasma) and dividing by 3.3, using the assumption that each haploid genomic equivalent weighs 3.3 pg, as previously described by Scherer et al(5).

Figure S1. Genetic subtype classification of the samples according to the LymphGen algorithm.

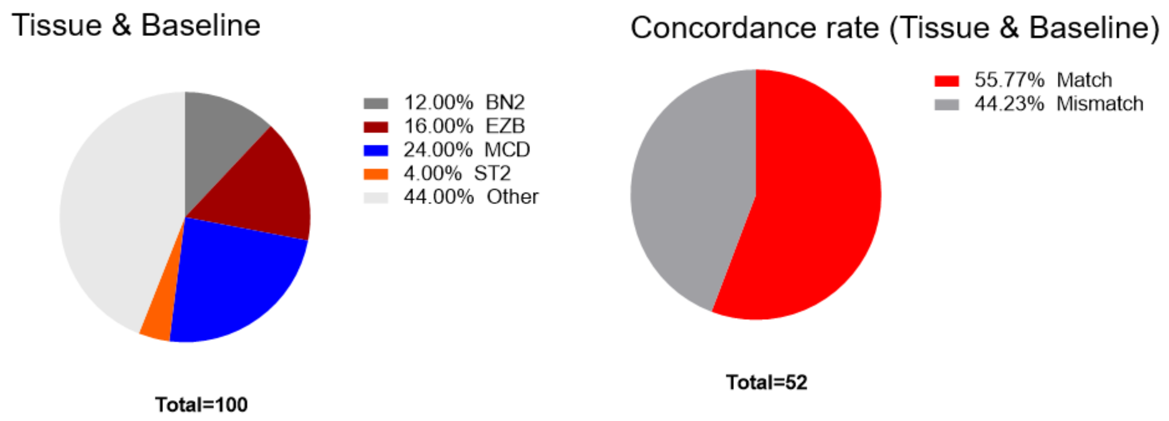

Figure S2. Baseline meanVAF (A), maxVAF (B), and median circulating tumor DNA concentration (C) difference according to the response to frontline immunochemotherapy.

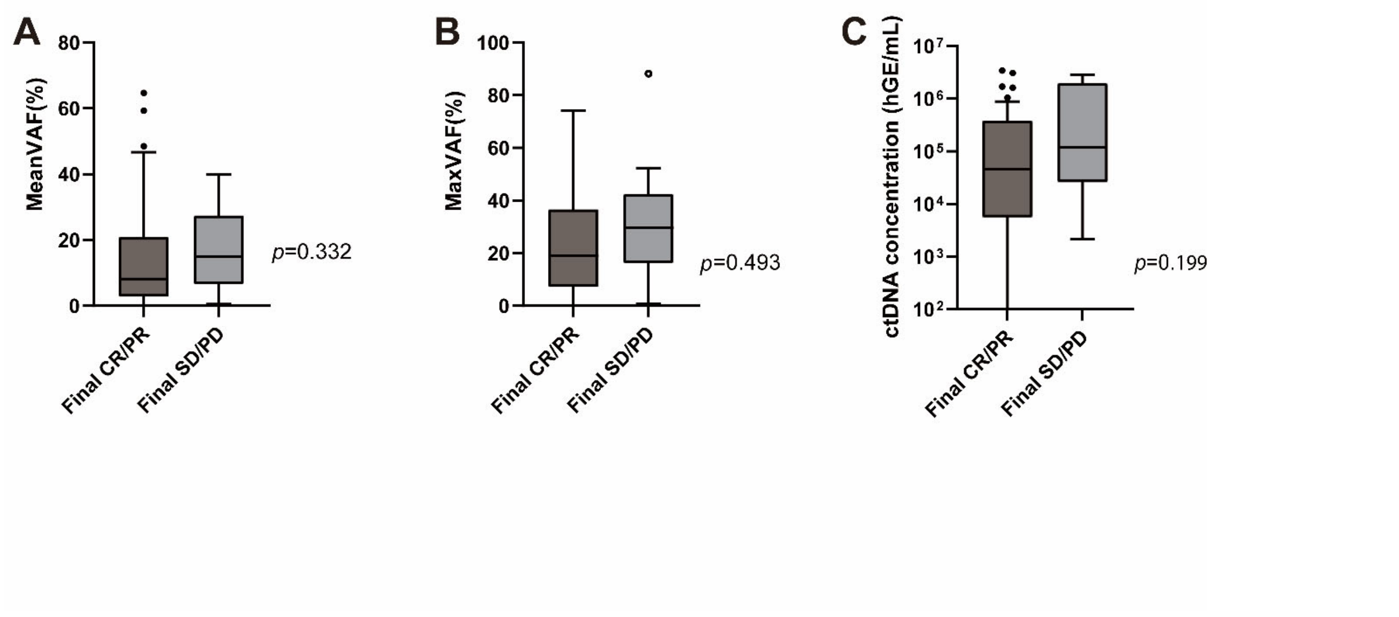

Figure S3. PFS (A) and OS (B) according to whether achieving more than 2log reduction of interim ctDNA or not.

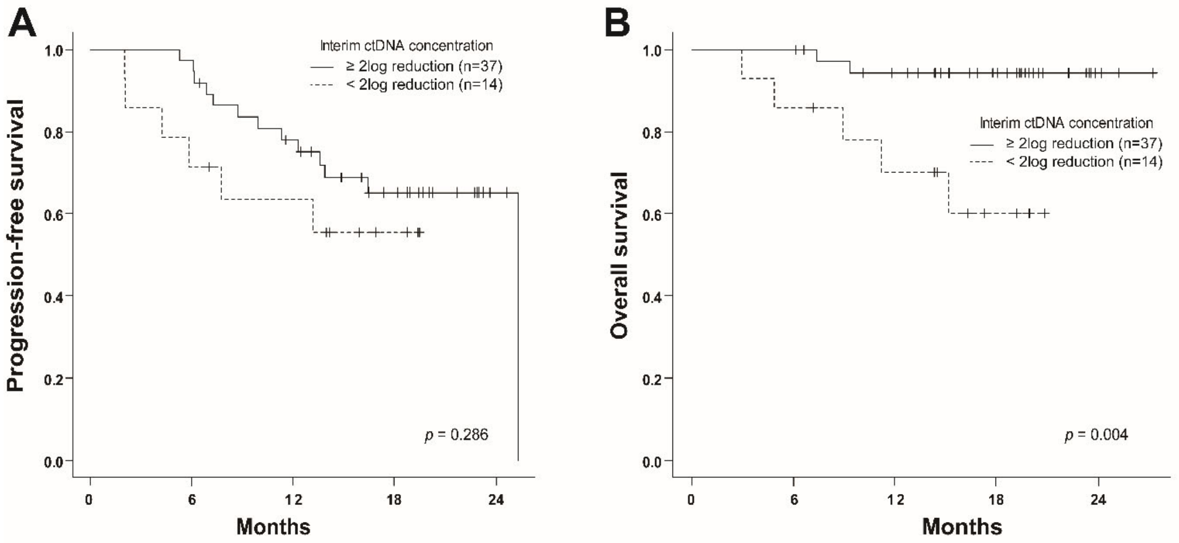

Figure S4. PFS (A) and OS (B) according to interim PET/CT and ctDNA combined response assessment. PFS (C) and OS (D) according to interim ctDNA response in patients who achieved PR in interim PET.

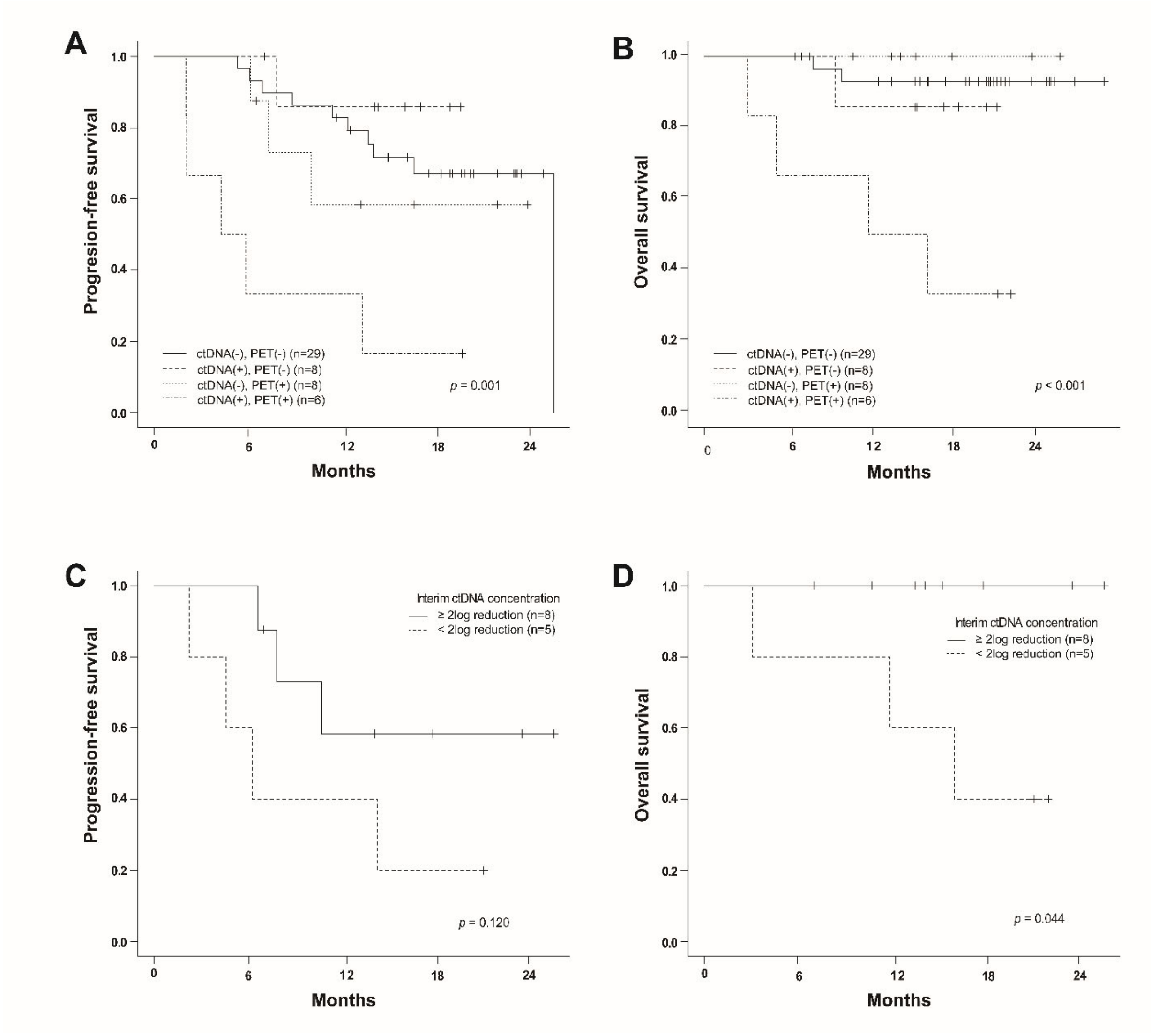
A
Progresion-free survival
Months
ctDNA(-), PET(-) (n=29)
ctDNA(+), PET(-) (n=8)
ctDNA(-), PET(+) (n=8)
ctDNA(+), PET(+) (n=6)
p = 0.001
B
Overall survival
Months
ctDNA(-), PET(-) (n=29)
ctDNA(+), PET(-) (n=8)
ctDNA(-), PET(+) (n=8)
ctDNA(+), PET(+) (n=6)
p < 0.001
C
Progression-free survival
Months
Interim ctDNA concentration
≥ 2log reduction (n=8)
< 2log reduction (n=5)
p = 0.120
D
Overall survival
Months
Interim ctDNA concentration
≥ 2log reduction (n=8)
< 2log reduction (n=5)
p = 0.044

Table S1. Genes included in the DxSeq Lymphoma & Myeloma panel

ALK	*ARID1A*	*ARID5B*	*ASXL1*	*ASXL3*	*ATM*	*B2M*
BCL11B	*BCL2*	*BCL6*	*BCOR*	*BCORL1*	*BIRC3*	*BIRC6*
BRAF	*BTG1*	*BTG2*	*BTK*	*CARD11*	*CCND1*	*CCND3*
CD28	*CD58*	*CD70*	*CD79A*	*CD79B*	*CDKN1B*	*CDKN2A*
CDKN2B	*CIITA*	*CREBBP*	*CXCR4*	*DDX3X*	*DIS3*	*DNMT3A*
DTX1	*DUSP22*	*EBF1*	*EGFR*	*EGR1*	*EP300*	*ETV6*
EZH2	*FAM46C*	*FAS*	*FBXW7*	*FOXO1*	*FYN*	*GNA13*
HIST1H1E	*ID3*	*IDH2*	*IKZF3*	*IRF4*	*IRF8*	*ITPKB*
JAK1	*JAK3*	*KDM6A*	*KIT*	*KLHL14*	*KLHL6*	*KMT2A*
KMT2C	*KMT2D*	*KRAS*	*LRP1B*	*MAP2K1*	*MEF2B*	*MGA*
MTOR	*MYC*	*MYD88*	*NCOR1*	*NF1*	*NFKB2*	*NFKBIA*
NOTCH1	*NOTCH2*	*NRAS*	*NSD2*	*PAX5*	*PHF6*	*PIM1*
PLCG1	*PRDM1*	*PTEN*	*RB1*	*REL*	*RHOA*	*SETD1B*
SETD2	*SF3B1*	*SGK1*	*SMARCA4*	*SOCS1*	*SPEN*	*STAT3*
STAT5B	*STAT6*	*TBL1XR1*	*TCF3*	*TET1*	*TET2*	*TET3*
TNFAIP3	*TNFRSF14*	*TP53*	*TP63*	*TRAF3*	*UBR5*	*XPO1*

Table S2. Dynamic pattern of *MYD88 L252P* and treatment response.

Sample	Tissue	Baseline	Interim	End-of-treatment	Final response
CHH-008	X	O	X	O	PD
CHH-010	O	O	X	X	CR
CHH-019	X	O	X	X	CR
CHH-026	O	O	X	X	CR
CHH-028	O	O	X	X	PD
CHH-030	X	O	X	X	CR
CHH-044	O	O	X	X	CR
CHH-047	X	O	X	X	CR
CHH-048	X	O	O	O	PD
CHH-049	X	O	X	X	CR

References

1. Li H. Toward better understanding of artifacts in variant calling from high-coverage samples. *Bioinformatics (Oxford, England)* 2014;**30**(20):2843-51 doi 10.1093/bioinformatics/btu356.
2. Rausch T, Zichner T, Schlattl A, Stütz AM, Benes V, Korbel JO. DELLY: structural variant discovery by integrated paired-end and split-read analysis. *Bioinformatics (Oxford, England)* 2012;**28**(18):i333-i9 doi 10.1093/bioinformatics/bts378.
3. Li MM, Datto M, Duncavage EJ, Kulkarni S, Lindeman NI, Roy S*, et al.* Standards and Guidelines for the Interpretation and Reporting of Sequence Variants in Cancer: A Joint Consensus Recommendation of the Association for Molecular Pathology, American Society of Clinical Oncology, and College of American Pathologists. *The Journal of molecular diagnostics : JMD* 2017;**19**(1):4-23 doi 10.1016/j.jmoldx.2016.10.002.

4. Richards S, Aziz N, Bale S, Bick D, Das S, Gastier-Foster J*, et al.* Standards and guidelines for the interpretation of sequence variants: a joint consensus recommendation of the American College of Medical Genetics and Genomics and the Association for Molecular Pathology. *Genetics in medicine : official journal of the American College of Medical Genetics* 2015;**17**(5):405-24 doi 10.1038/gim.2015.30.
5. Scherer F, Kurtz DM, Newman AM, Stehr H, Craig AF, Esfahani MS*, et al.* Distinct biological subtypes and patterns of genome evolution in lymphoma revealed by circulating tumor DNA. *Science translational medicine* 2016;**8**(364):364ra155 doi 10.1126/scitranslmed.aai8545.
